# Seed germination improves air classification efficiency of pea and faba bean flours

**DOI:** 10.1002/jsfa.70522

**Published:** 2026-02-12

**Authors:** Areha Abid, Kashika Sethi, Andrea K Stone, Xialu Chen, Ke Ding, Thomas D Warkentin, Bunyamin Tar'an, Yongfeng Ai, Michael T Nickerson

**Affiliations:** ^1^ Department of Food and Bioproduct Sciences University of Saskatchewan Saskatoon SK Canada; ^2^ Crop Development Centre and Department of Plant Sciences University of Saskatchewan Saskatoon SK Canada

**Keywords:** air‐classifier, pulse flour, protein‐rich stream, scanning electron microscope, soaking, starch‐rich stream

## Abstract

**BACKGROUND:**

Increasing interest in incorporating pulses into human diets has increased demand for their fractionation into diverse food ingredients. Air classification has relatively low capital and operating costs, uses no water, and preserves the native protein structure. However, its efficiency in separating protein and starch is lower than that of wet fractionation. This study investigated seed germination of pea and faba bean for 24, 48, and 96 h as a pretreatment to improve the air classification efficiency of pulse flours into fine (protein‐rich) and coarse (starch‐rich) streams.

**RESULTS:**

Scanning electron microscopy revealed that germination disrupted the protein and fiber matrices surrounding starch granules in pea and faba bean seeds, improving subsequent air classification efficiency. Following 24–96 h of soaking and germination, the fine stream yield increased from 251 to 295 g kg⁻¹ for pea and from 274 to 364 g kg⁻¹ for faba bean. Protein retention in the fine stream – the proportion of total protein recovered – also increased, from 620 to 686 g kg⁻¹ for pea and from 702 to 882 g kg⁻¹ for faba bean, both at 48 h of germination. After 96 h, protein retention declined. In contrast, germination had no effect on the starch retention in the coarse stream.

**CONCLUSION:**

In conclusion, 48 h of germination of pea and faba bean seeds is sufficient to improve the air classification efficiency of the resulting pulse flours. © 2026 The Author(s). *Journal of the Science of Food and Agriculture* published by John Wiley & Sons Ltd on behalf of Society of Chemical Industry.

## INTRODUCTION

Pulses, the dried, edible seeds of plants in the Leguminosae family, are an essential source of nutrients worldwide. They are rich in carbohydrates, proteins, dietary fibers, vitamins, minerals, and phytochemicals, making them an essential component of a balanced diet.^1^, ^2^ Processing pulses, such as lentils, peas, chickpeas, and faba beans, is a critical step in adding value to the raw commodities. Current pulse‐processing techniques include dry fractionation (air‐classification, tribo‐electric, or electrostatic separation) and wet fractionation, with solvent‐based wet fractionation being particularly effective at isolating protein.^3^

Air classification is cost‐effective, requires minimal water, and preserves native protein structures, but the separation efficiency for protein and starch is lower than that of wet fractionation.^3^ However, improvements in fractionation processes hold considerable potential for the value‐added pulse industry. Milling techniques, particle size of the feed material (flour), and the number of passes and parameters of the air classifier, have been the focus of much of the research on air classification separation efficiency to improve the yield and purity of the coarse (starch‐rich) and fine (protein‐rich) streams.^4^, ^5^, ^6^ Enhancing dry fraction techniques such as air classification to obtain higher yields, enhance the quality of pulse ingredients, and achieve more sustainable practices can promote the development of new, nutritious ingredients for human consumption, driving innovation and potentially increasing commodity prices.^7^, ^8^

Germination is known to improve the nutritional properties of pulses.^9^ Upon reaching specific sprout lengths, seeds attain maximal accumulation of digestible carbohydrates, proteins, folate, tocopherols, vitamin C, polyphenols, and *γ*‐aminobutyric acid (GABA), but the concentrations of enzyme inhibitors and other antinutrients, such as phytate, are reduced.^9^, ^10^ Previous studies have shown that germination disrupts the protein and fiber matrices surrounding starch granules in pulse seeds.^11^, ^12^ This effect is hypothesized to improve the air classification efficiency of pulse flours. Holopainen‐Mantila *et al*.^13^ reported that germination of faba bean improved protein separation in the air‐classified fine fraction, although the effect of germination time was not investigated.

The novelty of this study lies in using seed germination as a pretreatment to improve the air classification of protein‐ and starch‐rich streams derived from pea (CDC ‘Meadow’) and faba bean (DL ‘Rico’), a relatively unexplored approach in pulse processing. By examining how germination influences the dehulling efficiency of dried seeds and disrupts the protein and fiber matrices surrounding starch granules, the aim of this study was to enhance the efficiency of protein and starch separation during air classification of pin‐milled pea and faba bean flours. The results provide insights for optimizing pulse fractionation processes for value‐added applications in the food industry.

## MATERIALS AND METHODS

### Materials

Round pea (CDC ‘Meadow’) and faba bean (DL ‘Rico’) seeds (2020 harvest; dark brown soil zone, Floral, SK, Canada) were supplied by the Crop Development Centre, University of Saskatchewan (Saskatoon, SK, Canada). The Megazyme Total Starch Assay Kit, d‐Glucose Assay Kit, Starch Damage Assay Kit, and potato amylose standard for quantitating amylose content by iodine colorimetry were purchased from Neogen Corporation (Lansing, MI, USA). Sodium hypochlorite (reagent grade, 10% to 15%) for the sterilization of seeds and maize amylopectin standard used for amylose quantification were purchased from Sigma‐Aldrich Canada Co. (Oakville, ON, Canada). Other chemicals were reagent grade and acquired from either Sigma‐Aldrich Canada Co. or Fisher Scientific Company (Ottawa, ON, Canada).

### Soaking and germination of seeds

Pea and faba bean seeds were first washed with tap water to remove dust and other impurities. After washing, each batch of seeds (600 g) was then soaked in 1 L of distilled water with 0.07% (w/v) sodium hypochlorite (NaClO) for 30 min as a sterilization step to limit microbial growth during germination. The sterilized seeds were removed from the NaClO solution and then washed four or five times with distilled water and soaked in 1 L of distilled water overnight at room temperature (approximately 22 °C). These soaked seeds were designated as soaked (0 h germination) seeds in this study (two batches per pulse variety). Additional batches were germinated for 24, 48, and 96 h (two batches per pulse variety at each time point, 600 g per batch).

The soaked seeds were spread evenly in one layer on flat trays, covered with wet cheesecloth, and kept in dark conditions at room temperature. During germination, the seeds were sprayed once daily with approximately 50 g of distilled water to maintain adequate seed moisture. After the designated germination periods, samples were designated as 24, 48, and 96 h germinated. Germination percentage at each time point was calculated by dividing the number of germinated seeds by the total number of seeds in the batch. Radicle length was measured for ten seeds randomly selected from each batch using a Vernier caliper at the end of each germination period. Seeds from the soaked and germinated batches were dried in a convection oven (Binder FD 56, Binder GmbH, Tuttlingen, Germany) at 50 °C until the moisture content was reduced to 70–90 g kg^−1^ (approximately 24 h). Control samples consisted of raw, untreated seeds with moisture content of 70–90 g kg^−1^.

### Dehulling of raw, soaked, and germinated pulse seeds

The raw (control), soaked, and germinated seeds (24, 48, and 96 h) were dehulled using a bench‐scale dehuller (106B, Canadian General Electric, Peterborough, ON, Canada). After dehulling, the yield of dehulled seeds was calculated using the following equation:

$$\text{yield of dehulled seeds}=\frac{\text{weight of dehulled seeds}}{\text{initial weight of whole seeds}}$$

### Pin milling of raw, soaked, and germinated pulse seeds and air classification of resulting flours

The dehulled raw (control), soaked, and germinated (24, 48, and 96 h) pulse seeds were milled into flours using a two‐step process: (1) a ‘pre‐break’ step: the seeds were milled into coarse grits using a laboratory disc mill (Model 3310, Perten Instruments AB, Sweden); and (2) a ‘pin‐mill’ step: the coarse grits were further milled using a pin mill (Model 100 UPZ, Hosokawa Alpine, Augsburg, Germany) at 22 000 rpm to prepare finer flours. A portion of each pin‐milled pulse flour was collected and labeled as ‘milled flour’ for chemical composition analysis, and the remaining pin‐milled pulse flour was fractionated using an air classifier (Model 100 MZR, Hosokawa Alpine, Augsburg, Germany) at Cereals Canada (Winnipeg, MB, Canada) according to a previously described method.^7^ Approximately 500 g of each pin‐milled flour was air classified every hour at an air classifier wheel speed of 12 000 rpm and an air‐flow rate of 46 m³ h⁻¹. After the air classification, two streams were obtained from each batch of processing, namely a fine (protein‐rich) fraction and a coarse (starch‐rich) fraction.

### Morphology of seeds and air‐classified fine and coarse streams

The structure and the morphology of the raw (control), soaked, and germinated (48 h only) pulse seeds and the air‐classified fine and coarse streams were observed using a scanning electron microscope (SEM) (SU8010, Hitachi High Technologies Canada Inc., Rexdale, ON, Canada). The seeds were carefully broken into smaller pieces manually. After coating the exposed seed cross section and the air‐classified powders with gold (10 nm thickness) using the Q150T ES coater, the samples were examined under the SEM at three magnifications: 30×, 500×, and 1000×.

### Particle‐size distributions of fine and coarse streams

Particle‐size distributions of the pin‐milled flour and fine and coarse streams were determined using Malvern Scirocco 2000 Mastersizer (Malvern Panalytical, Saint‐Laurent, QC, Canada). Briefly, the flour/powder (approximately 2 g) was suspended in 20 mL of distilled water under magnetic stirring at 250 rpm for 5 min. The flour suspension was then loaded to the dispersion cell dropwise using a disposable pipette. The particle‐size distribution and volume‐weighted mean particle size (D[3, 4]) were recorded by Mastersizer 2000 Version 5.54 Software (Malvern Panalytical) after the laser obscuration reading fit into a range of 10% to 20%.

### Chemical compositions and separation efficiency of fine and coarse streams

The protein, ash, and starch content of the milled flour and fine and coarse streams was determined. Lipid content was determined for the milled flour only by extraction with petroleum ether using the Goldfish apparatus following the AOAC 920.85 method.^14^ Nitrogen content was measured using a LECO FP628 nitrogen determinator (LECO Corp., St. Joseph, MI, USA) and converted to protein content (%N × 6.25) using AACC method 46–30.01.^15^ Ash content was determined following the AACC 08‐01.01 method.^15^ The Megazyme Total Starch Assay Kit was used to determine total starch content following AACC Method 76–13.01.^15^ Damaged‐starch content was determined for the coarse stream only using the Megazyme Starch Damage Assay Kit following AACC Method 76‐31.01.^15^ Amylose content was also determined for the coarse stream only in accordance with an iodine colorimetric method^16^ given in detail by Li *et al*.^17^ Protein and starch retentions in the fine and coarse streams, respectively, were calculated by:

$$\begin{array}{l} \text{protein retention in fine stream}\\ \;=\frac{\text{yield}\;\text{of fine stream from}\;\mathrm{air}\;\text{classification}\;\times \;\text{protein content}\;\text{of fine stream}\left(\mathrm{dry}\;\text{basis}\right)}{\text{protein content}\;\text{of}\;\mathrm{pin}\;-\;\text{milled flour}\left(\mathrm{dry}\;\text{basis}\right)} \end{array}$$

$$\begin{array}{l} \text{starch retention in coarse stream}\\ \;=\frac{\text{yield}\;\text{of coarse stream from}\;\mathrm{air}\;\text{classification}\;\times \;\text{starch content of}\;\text{coarse stream}\left(\mathrm{dry}\;\text{basis}\right)}{\text{starch content}\;\text{of}\;\mathrm{pin}\;-\;\text{milled flour}\left(\mathrm{dry}\;\text{basis}\right)} \end{array}$$

### Statistical analysis

Germination, milling, and air classification of the pulses were conducted in two independent batches. For each batch of samples, all the analyses were conducted in duplicate unless specifically indicated. The data were reported as means ± standard deviations. Statistical differences were determined by one‐way analysis of variance (ANOVA) with Tukey's honestly significant difference (HSD) test at a significance level of 0.05 using IBM SPSS Software Version 25 (IBM Corporation, Armonk, NY, USA).

## RESULTS AND DISCUSSION

### Germination percentage, radicle lengths, mass recovery and dehulling efficiency of pulse seeds

During the 96 h germination period, the germination percentages ranged from 89.2% to 91.2% for pea and from 93.6% to 95.4% for faba bean (Table 1), indicating high seed viability. Pea radicle length increased from 7.3 mm at 24 h to 49.2 mm at 96 h, substantially greater than that of faba bean (6.3–21.5 mm) over the same germination period, suggesting a faster pea germination rate, as also reported by Setia *et al*.^12^ Mass recovery of pea ranged from 863 to 960 g kg^−1^, whereas that of faba bean ranged from 920 to 963 g kg^−1^ during the 96 h germination period.

**Table 1:** Germination percentages, radicle length, dry mass recovery, and dehulling efficiency of pea (CDC ‘Meadow’) and faba bean (DL ‘Rico’)

Sample	Germination percentage (%)	Radicle length (mm)	Dry mass recovery after germination (g kg^−1^)	Dehulling efficiency (g kg^−1^)
Pea				
Control (raw)	‐	‐	‐	842 ± 25^c^
Soaked	‐	‐	970 ± 5^b^	883 ± 3^e^
24 h germinated	89.2 ± 0.4^a^	7.3 ± 0.4^a^	960 ± 4^b^	828 ± 67^b^
48 h germinated	91.1 ± 0.3^b^	27.6 ± 0.1^b^	952 ± 3^b^	860 ± 44^d^
96 h germinated	91.2 ± 0.6^b^	49.2 ± 0.5^c^	863 ± 13^a^	787 ± 15^a^
Faba bean				
Control (raw)	‐	‐	‐	790 ± 14^ab^
Soaked	‐	‐	971 ± 2^c^	790 ± 4^ab^
24 h germinated	93.6 ± 0.1^a^	6.3 ± 0.1^a^	963 ± 5^c^	804 ± 4^b^
48 h germinated	95.3 ± 0.2^b^	9.4 ± 0.5^b^	951 ± 6^b^	798 ± 7^b^
96 h germinated	95.4 ± 0.2^b^	21.5 ± 3.7^c^	920 ± 4^a^	761 ± 24^a^

Values are presented as mean averages ± standard deviations (*n* = 2). Numbers with the same letter in the same column for each pulse type are not significantly different at *P* < 0.05.

Germination percentage was calculated by dividing the number of germinated seeds over the total number of seeds of one batch.

Radicle length was determined for ten randomly selected seeds in one batch; measurement was carried out using a Vernier caliper.

A gradual decrease in mass recovery was observed with increasing germination duration. This is consistent with previous studies showing that seed mass decreases during germination as stored nutrient reserves are broken down and utilized.^18^, ^19^, ^20^, ^21^ The dehulling efficiency of faba bean remained unchanged after soaking and up to 48 h germination but decreased after 96 h. Similarly, the dehulling efficiency of pea declined at 96 h germination. This decrease is likely due to lower seed density and reduced cotyledon integrity, causing a larger proportion of small, light cotyledon particles to be lost with the hull during dehulling. Soaking, however, increased pea dehulling efficiency from 842 to 883 g kg^−1^. Similar improvements after soaking have been reported for other pulses and are attributed to cotyledon swelling and subsequent contraction, which enhances separation from the hull.^22^, ^23^

### Chemical composition of pin‐milled pulse flours

Table 2 reports the composition of the pin‐milled pulse flours. The pin‐milled samples of pea and faba bean showed that protein content increased gradually with increasing germination time. For both pulses, the protein content did not increase until 48 h germination. The protein content increased slightly in pea, from 250 g kg^−1^ (control) to 273 g kg^−1^ for 96 h germination. This improvement was more pronounced in faba bean as the protein content increased from 289 g kg^−1^ (control) to 345 g kg^−1^ over the same germination time period. Germination causing an increase in the protein content of pulses has been reported previously and is associated with protein synthesis and hydrolysis of previously unavailable protein.^11^, ^24^ The crude fat content of the pin‐milled pea flours increased proportionally to the protein content, whereas for faba bean the highest crude fat content was for the 48 h germinated sample. There were only minor differences in the starch content of raw, soaked, and germinated pea with values of approximately 540–552 g kg^−1^. However, for faba bean, the starch content decreased from 511 g kg^−1^ (control) to 472 g kg^−1^ after 48 h germination. This breakdown of starch during germination may also partially explain the higher protein content of germinated faba bean, as the decrease in one nutrient results in a relative mass balance increase of another nutrient. The pea flour ash content decreased from 27 g kg^−1^ to 22 g kg^−1^ after soaking, whereas after 96 h germination it was 25 g kg^−1^.

**Table 2:** Chemical compositions of pin‐milled pea (CDC ‘Meadow’) and faba bean (DL ‘Rico’) flours on a dry basis

Sample	Protein (g kg^−1^)	Starch (g kg^−1^)	Crude fat (g kg^−1^)	Ash (g kg^−1^)
Pea				
Control (raw)	250 ± 1^a^	549 ± 5^b^	11 ± 1^a^	27 ± 0^d^
Soaked	246 ± 0^a^	542 ± 3^b^	11 ± 0^a^	22 ± 0^a^
24 h germinated	246 ± 0^a^	552 ± 6^c^	11 ± 1^a^	22 ± 0^a^
48 h germinated	266 ± 1^b^	540 ± 5^a^	14 ± 1^b^	23 ± 0^b^
96 h germinated	273 ± 3^c^	547 ± 7^b^	16 ± 1^c^	25 ± 0^c^
Faba bean				
Control (raw)	289 ± 3^ab^	511 ± 4^d^	8 ± 1^a^	28 ± 1^a^
Soaked	280 ± 5^a^	513 ± 3^d^	8 ± 0^b^	25 ± 1^a^
24 h germinated	295 ± 3^b^	495 ± 5^c^	8 ± 1^b^	26 ± 0^a^
48 h germinated	324 ± 0^c^	472 ± 4^a^	14 ± 0^d^	27 ± 0^a^
96 h germinated	345 ± 1^d^	477 ± 6^b^	11 ± 1^c^	28 ± 3^a^

Values are presented as mean averages ± standard deviations (*n* = 4; duplicate measurements of two batches). Numbers with the same letter in the same column for each pulse type are not significantly different at *P* < 0.05.

This could be attributed to the leaching of minerals during soaking and germination. Germination is known to activate enzymatic activity, which hydrolyzes stored nutrients in the seed, resulting in the release of minerals and other nutrients.^24^ This process could have contributed to the decrease in ash content in the 24 h germinated samples because some minerals could have been mobilized and utilized during the early stages of germination. The constant ash content in the 48 h germinated samples suggests that the nutrient mobilization process might have slowed or ceased, resulting in stable ash content. However, the significant increase in ash content in the 96 h germinated samples could be attributed to the accumulation of minerals and other nutrients during the later stages of germination. The prolonged germination duration might have allowed for more extensive nutrient mobilization and absorption, leading to increased ash content. In contrast, soaking and germination did not affect the faba bean ash content significantly. Other components that can be present in the flours are non‐starch carbohydrates, dietary fiber and minor constituents like vitamins, minerals and phytochemicals. These findings are consistent with previous studies that have reported changes in the nutritional composition of legumes after soaking and germination.^11^, ^25^

### Yield of fine and coarse streams of air classified pulse flours

The total yield of the two streams (fine and course) from air classification exceeded 970 g kg^−1^ for each pulse type regardless of treatment, indicating efficient recovery during the process (Table 3). Soaking did not affect pea fine stream yield, but germination increased it gradually from 257 g kg^−1^ at 24 h germination to 295 g kg^−1^ at 96 h. For faba bean, the fine stream yield increased after soaking from 274 g kg^−1^ for the control (raw seeds), to 333 g kg^−1^, with a further increase to 373 g kg^−1^ after 48 h germination; extending germination to 96 h provided no additional benefit. Coarse stream yield decreased proportionately for each pulse: 739–701 g kg^−1^ for pea and 715–630 g kg^−1^ for faba bean (Table 3). These results indicate that the germination pretreatments produced a larger quantity of fine stream. Similarly, Holopainen‐Mantila *et al*.^13^ reported that fine stream yield of faba bean increased from 275 to 334 g kg⁻¹ after germination, with a corresponding decrease in coarse stream yield from 688 to 574 g kg⁻¹.

**Table 3:** Fine and coarse stream yields of air‐classified pea (CDC ‘Meadow’) and faba bean (DL ‘Rico’)

Sample	Fine stream yield (g kg^−1^)	Coarse stream yield (g kg^−1^)	Total yield (g kg^−1^)
Pea			
Control (raw)	251 ± 3^a^	739 ± 1^b^	991 ± 1^a^
Soaked	249 ± 3^a^	727 ± 7^ab^	976 ± 4^a^
24 h germinated	257 ± 5^a^	717 ± 4^ab^	973 ± 10^a^
48 h germinated	277 ± 5^b^	717 ± 11^ab^	994 ± 6^a^
96 h germinated	295 ± 17^bc^	701 ± 13^a^	996 ± 4^a^
Faba bean			
Control (raw)	274 ± 17^a^	715 ± 20^b^	989 ± 3^a^
Soaked	323 ± 3^b^	674 ± 1^ab^	997 ± 2^a^
24 h germinated	326 ± 2^bc^	668 ± 20^ab^	994 ± 17^a^
48 h germinated	373 ± 2^d^	620 ± 1^a^	992 ± 2^a^
96 h germinated	364 ± 13^cd^	630 ± 14^a^	994 ± 1^a^

Values are presented as mean averages ± standard deviations (*n* = 2). Numbers with the same letter in the same column for each pulse type are not significantly different at *P* < 0.05.

### Morphology of pulse seed cotyledons and respective air‐classified fine and coarse streams

**Figure 1:**
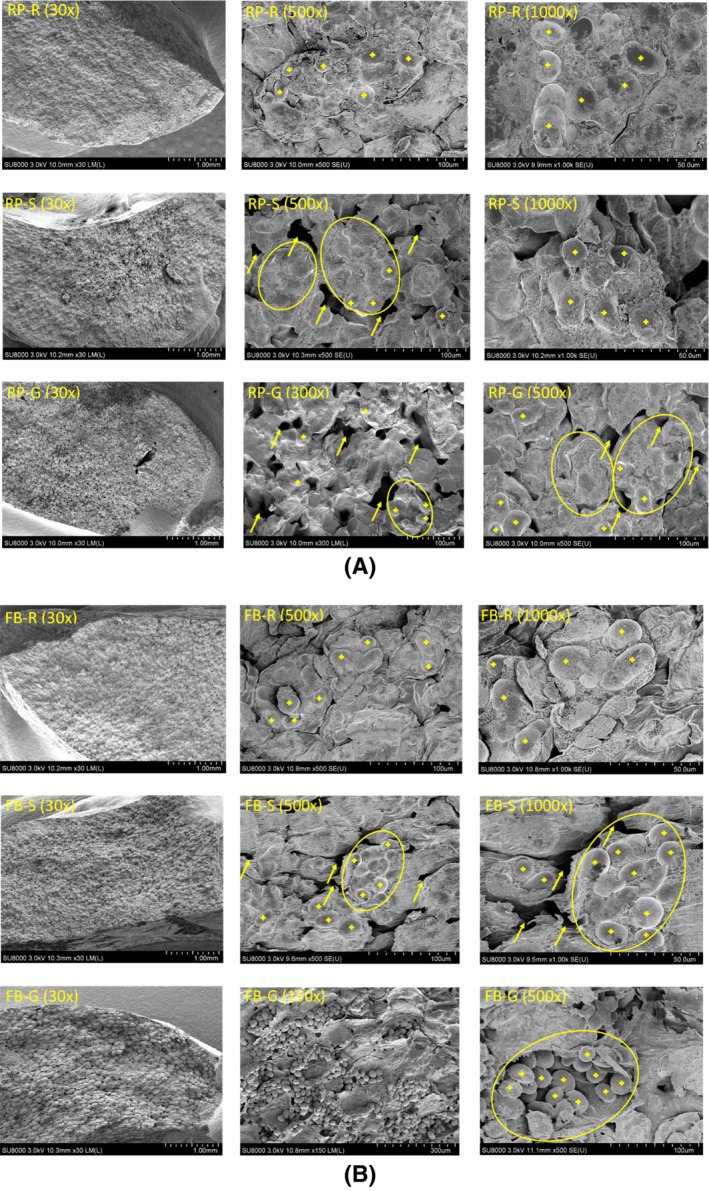
Scanning electron microscopy (SEM) images of cross sections of cut cotyledons of (A) round pea (RP) and (B) faba bean (FB). R: raw; S: soaked; and G: 48 h germinated. Magnification used for image capture is provided in parentheses. Plus (+) symbols indicate starch granules; arrows indicate spaces in the protein and fiber matrix; ovals indicate unruptured cotyledon matrix.

Figure 1 shows the morphology of the cross section of raw, soaked, and 48 h germinated pea and faba bean cotyledons. The SEM images of raw pea and faba bean indicate that the starch granules (indicated by plus symbols) were compactly embedded in the protein and fiber matrix in intact cells (indicated by ovals). Soaking loosened the matrix, liberating some starch granules and creating voids (indicated by arrows) between cotyledon cells. During soaking, the pulse seeds absorbed water (reaching a moisture content of approximately 570 g kg^−1^, data not shown) and swelled, physically disrupting the matrix structure.^26^ Germination further loosened the matrices of both pulses (as indicated by arrows) and thus more starch granules were released from the structure. The additional rupture of the matrix structure is attributed to the breakdown of protein and fiber components such as cellulose, hemicellulose, and pectin, by their respective hydrolytic enzymes.^12^, ^27^, ^28^ Hydrolysis of granular starch did not appear to occur during germination as no obvious changes were observed in the surface structure or morphology of the starch granules (Fig. 1).

As expected, the fine and coarse streams of pea and faba bean obtained after air classification displayed different morphologies under SEM. Grinding the cotyledons released starch granules from the cellular fiber matrix. Pea and faba bean starch granules can be recognized as smooth, spherical or oval particles, whereas the fragments of different sizes and irregular shapes correspond to protein and fiber particles. No differences within the fine fractions or coarse fractions from raw, soaked, or germinated seeds were apparent from the SEM images.

### Particle‐size distribution of pin‐milled pulse flours and air‐classified fine and coarse streams

Particle size distribution (PSD) curves can indicate the efficiency of starch separation from the protein matrix and the cell wall. To test the hypothesis that germination as a seed pretreatment improves air classification of pin‐milled pea and faba bean flours, PSD curves were analyzed, with longer germination expected to have a greater impact. The PSD of the pin‐milled flours and the fine and coarse streams obtained from air classification are shown in Fig. 3 (pea) and Fig. 4 (faba bean).

For all pin‐milled pea flours the PSD curves were similar in shape. The D[3, 4] of the flours from the control and 24 h germinated seeds were similar (26.9 μm), whereas the flour from soaked seeds had the smallest particle size (25.0 μm), and the flour from the 48 h germinated seeds had the largest particle size (27.8 μm). The fine stream fraction from pea followed a similar trend and increased in particle size as germination time increased up to 48 h (from 18.3 μm to 24.3 μm).

For faba bean, the control pin‐milled flour PSD curve was distinct from the other samples and had the largest D[3, 4] (37.8 μm), whereas the soaked and 24 h and 48 h germinated samples were all similar at approximately 30 μm. The pea coarse stream had D[3, 4] values ranging from 34.5 to 38.6 μm, whereas for faba bean it was 37.8–46.3 μm. The coarse stream PSD curve of raw (control), soaked, and 24 h germinated pea showed a small peak of fine particles (<10 μm), indicating inefficient separation during air classification. After 48 and 96 h germination the PSD curves of the pea coarse stream were monomodal and did not exhibit such peaks indicating improved separation efficiency especially for the 48 h germinated coarse stream, which had a significantly larger particle size than the other samples. Similar results were found for the faba bean coarse streams; however, only the control had a small peak below 10 μm, whereas the soaked and germinated samples did not. Despite this small peak of fine particles, the D[3, 4] for the coarse streams was highest for the control (46.3 μm).

### Chemical composition and protein retention of fine stream

The protein content of the fine stream of soaked, 24 and 48 h germinated pea and faba bean showed a gradual increase with increasing germination duration (Table 4). For pea, protein content increased from 616 g kg⁻¹ in the control to 659 g kg⁻¹ after 48 h germination, whereas for faba bean it increased from 742 to 766 g kg⁻¹ over the same period. This increase can be attributed to the higher protein content of the flour after 48 h germination and to microstructural disruption that enhances separation of starch and protein particles during air classification.^29^ However, longer germination (96 h) did not increase the protein content and, in fact, decreased the protein content of the fine stream to 609 g kg^−1^ for pea and 728 g kg^−1^ for faba bean, which may indicate that the optimal separation of protein and starch occurred within the first 48 h of germination. The concentration of protein in the fine stream has been well documented in the literature.^30^, ^31^, ^32^, ^33^, ^34^, ^35^, ^36^ The yield (Table 3) and purity of streams resulting from air classification were interrelated, with higher stream yields associated with lower purity in the 96 h germinated samples. This trend is consistent with previous reports.^36^, ^37^, ^38^

**Table 4:** Chemical composition and protein retention in the fine stream on a dry basis

Sample	Protein retention (g kg^−1^)	Protein (g kg^−1^)	Starch (g kg^−1^)	Ash (g kg^−1^)
Pea				
Control (raw)	620 ± 8^a^	616 ± 13^a^	32 ± 1^a^	50 ± 1^d^
Soaked	681 ± 9^c^	673 ± 9^b^	29 ± 2^a^	41 ± 1^b^
24 h germinated	685 ± 14^c^	657 ± 3^b^	29 ± 2^a^	42 ± 1^b^
48 h germinated	686 ± 13^c^	659 ± 3^b^	30 ± 1^a^	46 ± 1^c^
96 h germinated	658 ± 39^b^	609 ± 9^a^	53 ± 4^b^	36 ± 1^a^
Faba bean				
Control (raw)	702 ± 43^a^	742 ± 15^ab^	15 ± 1^a^	55 ± 1^d^
Soaked	837 ± 8^bc^	777 ± 9^c^	20 ± 1^ab^	48 ± 1^b^
24 h germinated	825 ± 4^bc^	747 ± 7^ab^	18 ± 1^a^	49 ± 0^c^
48 h germinated	882 ± 4^c^	766 ± 7^bc^	31 ± 3^bc^	50 ± 1^c^
96 h germinated	767 ± 27^ab^	728 ± 17^a^	41 ± 9^c^	46 ± 1^a^

Values are presented as mean averages ± standard deviations (*n* = 4) on a dry basis. Numbers with the same letter in the same column for each pulse type are not significantly different at *P* < 0.05.

Protein retention in fine stream = (yield of fine stream from air classification × protein content of fine stream, dry basis)/(protein content of pin‐milled flour, dry basis).

**Figure 2:**
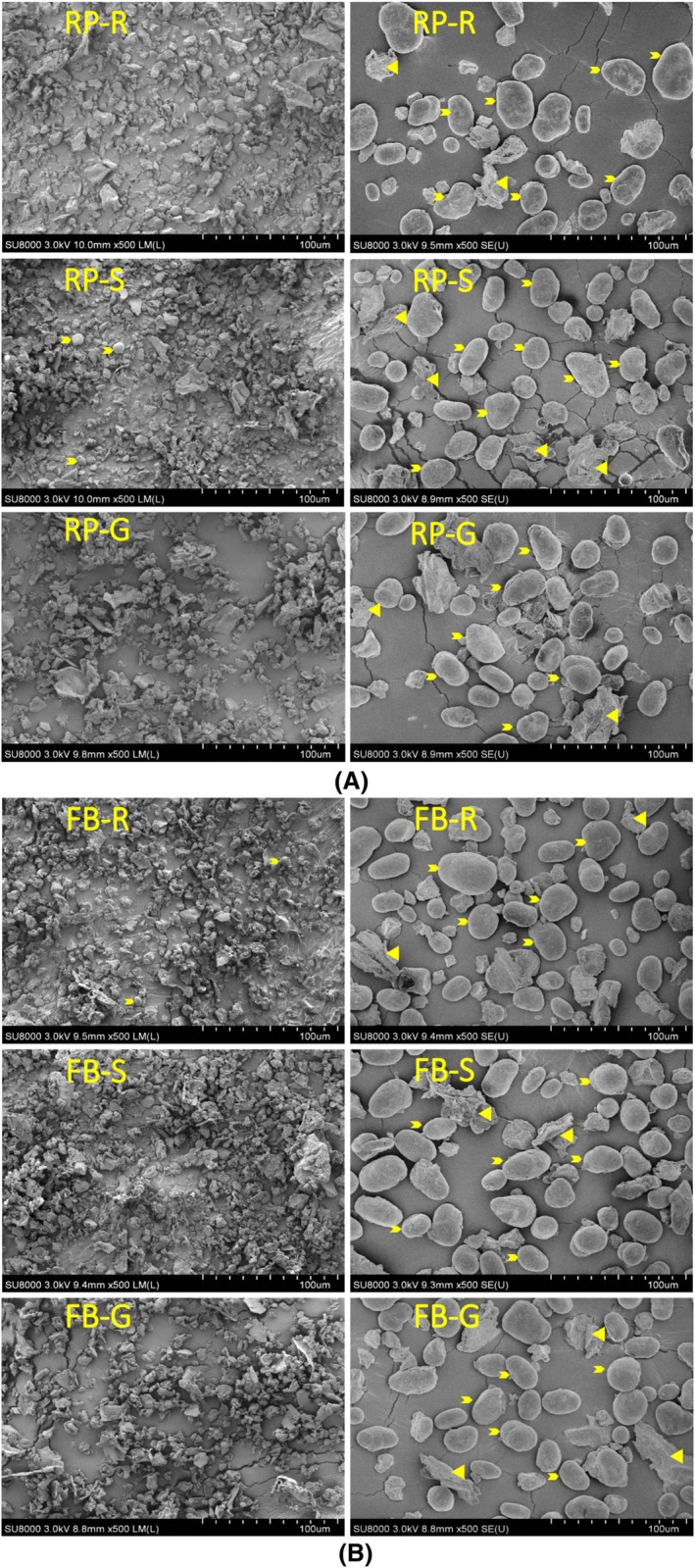
Scanning electron microscopy (SEM) images of fine and coarse streams of (A) round pea (RP) (CDC ‘Meadow’) and (B) faba bean (FB) (DL ‘Rico’). R: raw; S: soaked; and G: 48 h germinated. Magnification used for image capture is provided in parentheses. Small arrows indicate starch granules; triangles indicate protein/fiber particles.

Protein retention in the fine stream followed a similar trend to that of protein content (Table 4). Germination for 48 h markedly increased the amount of protein retention in the fine stream, from 620 g kg^−1^ to 686 g kg^−1^ for pea and from 702 g kg^−1^ to 882 g kg^−1^ for faba bean. Extending germination to 96 h reduced these values; however, values remained higher than the control for both pulses. As Figs 1 and 2 show, germination for 24 and 48 h partially disrupted the cotyledon matrix of pea and faba bean, facilitating the release of starch granules from the compact protein‐ and cell‐wall fiber‐rich cellular structure during milling and subsequent air classification. However, prolonged germination for 96 h likely caused excessive disruption of the protein and fiber matrix, reducing the particle sizes of protein and fiber to levels unfavorable for milling and air classification.

**Figure 3:**
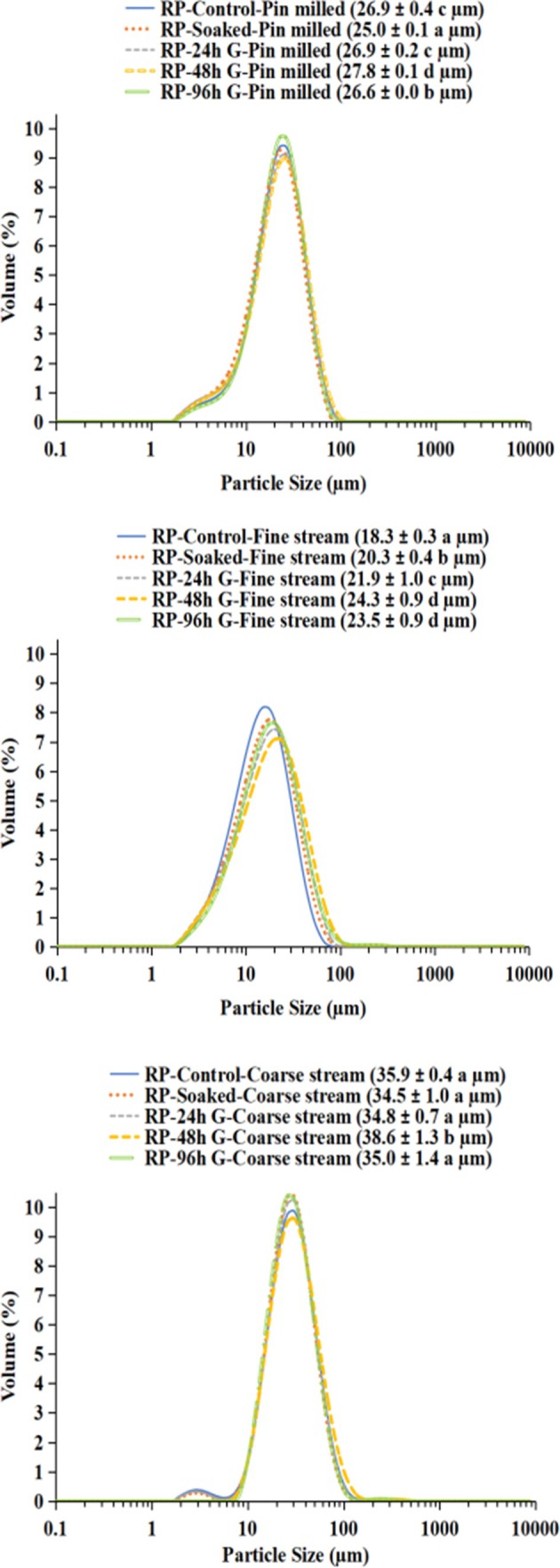
Particle‐size distributions of pin‐milled flours (top), fine streams (middle) and coarse streams (bottom) of round pea (CDC ‘Meadow’). D[3, 4] are presented in brackets as average ± standard deviation; within each graph lower case letters represent significant differences (*P* < 0.05; *n* = 4).

**Figure 4:**
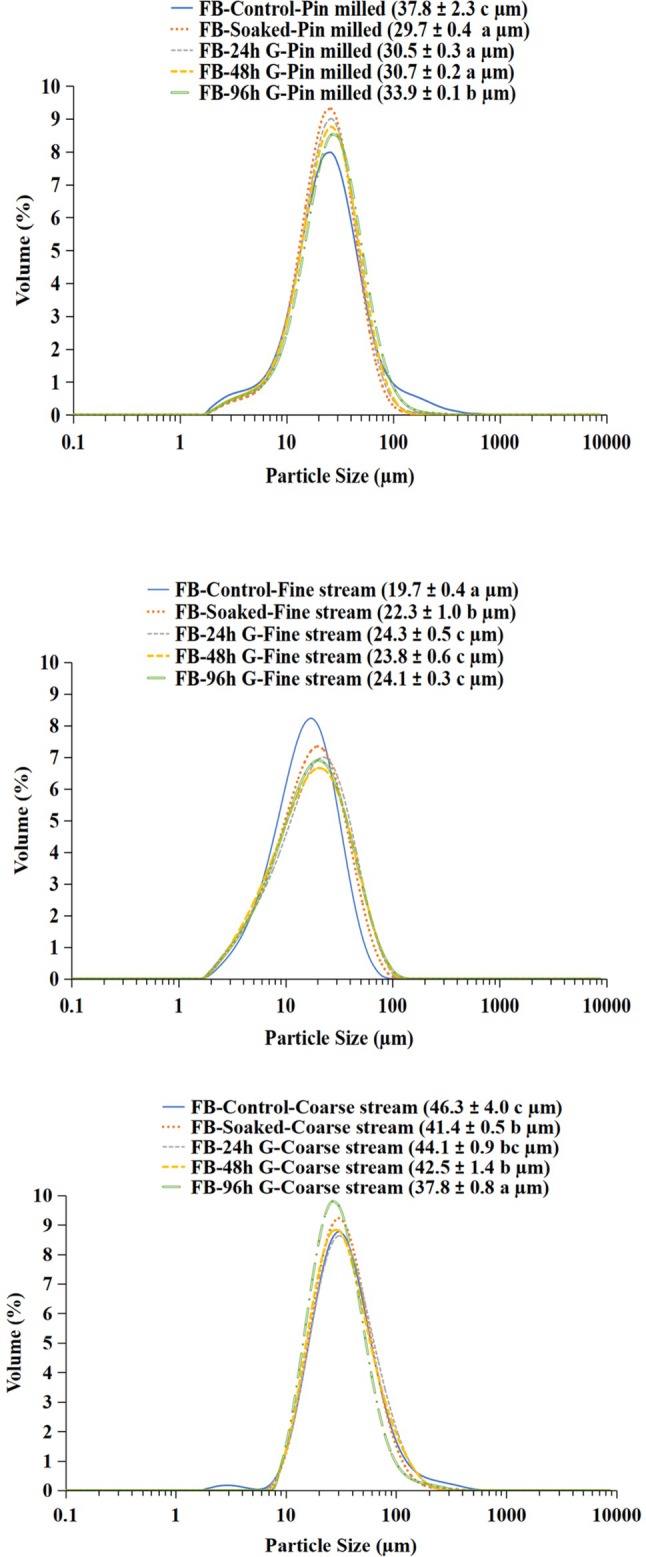
Particle‐size distributions of pin‐milled flours (top), fine streams (middle) and coarse streams (bottom) of faba bean (DL ‘Rico’). D[3, 4] are presented in brackets as average ± standard deviation; within each graph lower case letters represent significant differences (*P* < 0.05; *n* = 4).

The ash content of the fine streams decreased with soaking and germination for both pulse types (Table 4). For pea, ash content decreased from 50 to 36 g kg^−1^ after 96 h germination, whereas for faba bean it decreased from 55 to 46 g kg^−1^. Despite this reduction, ash content in the fine streams remained higher than the original pulse flours, which is expected because ash concentrates with protein during air classification.^34^, ^35^ These changes may have important implications for the use of pulse fractions in food products, as ash content can impact the nutritional value of the final product.

### Chemical composition and starch retention of coarse stream

Table 5 reports the chemical composition and starch retention in the coarse stream. Starch retention in the coarse streams (>900 g kg^−1^) was higher than the protein retention in the fine streams, which is consistent with previous work showing that air classification increased the starch content of pulse flours in the coarse stream and decreased it in the fine stream.^39^, ^40^ The starch content of the coarse streams of pea and faba bean increased with soaking and increasing germination duration up to 48 h indicating improved separation of starch from protein–fiber matrix during air classification. For pea, this increase was from 702 to 734 g kg^−1^ whereas for faba bean it increased from 688 to 738 g kg^−1^. Improved separation of protein and starch with increasing germination time therefore contributed to the observed increase in starch content. Germination tends to loosen the protein–fiber matrix, as also seen in the SEM images, facilitating improved protein and starch separation during air‐classification. Overall, germination did not substantially alter the damaged starch or amylose content of pea and faba bean coarse streams (Table 5), consistent with previous findings.^12^

**Table 5:** Chemical composition and starch retention in the coarse stream on a dry basis

Sample	Starch retention (g kg^−1^)	Protein (g kg^−1^)	Starch (g kg^−1^)	Damaged starch (g kg^−1^)	Amylose (g kg^−1^, dry flour basis)	Amylose (g kg^−1^, dry starch basis)	Ash (g kg^−1^)
Pea							
Control (raw)	946 ± 3^a^	126 ± 8^a^ ^b^	702 ± 9^a^	23 ± 2^a^ ^b^	271 ± 7^a^	387 ± 6^ab^	14 ± 1^a^
Soaked	950 ± 9^a^	131 ± 1^a^ ^b^	708 ± 5^a^	25 ± 1^b^ ^c^	274 ± 6^a^	388 ± 7^ab^	14 ± 0^a^
24 h germinated	929 ± 6^a^	128 ± 3^a^ ^b^	716 ± 9^a^	24 ± 1^b^	287 ± 6^b^	401 ± 11^b^	13 ± 1^a^
48 h germinated	974 ± 15^a^	123 ± 2^a^	734 ± 3^b^	22 ± 1^a^	278 ± 3^ab^	378 ± 3^a^	14 ± 1^a^
96 h germinated	953 ± 18^a^	132 ± 2^b^	743 ± 10^b^	27 ± 1^c^	283 ± 5^ab^	381 ± 10^a^	18 ± 0^b^
Faba bean							
Control (raw)	963 ± 26^a^	193 ± 3^c^	688 ± 6^a^	30 ± 1^c^	256 ± 3^a^	373 ± 8^b^	17 ± 0^b^
Soaked	910 ± 2^a^	164 ± 1^b^	693 ± 4^a^	28 ± 1^bc^	256 ± 4^a^	370 ± 5^b^	14 ± 1^a^
24 h germinated	938 ± 27^a^	155 ± 2^ab^	695 ± 2^a^	27 ± 1^ab^	249 ± 4^a^	358 ± 5^b^	14 ± 1^a^
48 h germinated	969 ± 1^a^	151 ± 2^a^	738 ± 4^c^	25 ± 1^a^	251 ± 6^a^	340 ± 8^a^	14 ± 1^a^
96 h germinated	956 ± 21^a^	156 ± 11^ab^	724 ± 4^b^	29 ± 2^c^	247 ± 5^a^	341 ± 8^a^	16 ± 1^b^

Values are presented as average ± standard deviation (*n* = 4; duplicate measurements of two batches) on a dry basis, the numbers with the same letter in the same column for each pulse type are not significantly different at *P* < 0.05.

Amylose content, dry starch basis = (amylose content, dry flour basis)/(starch content, dry flour basis).

Starch retention in coarse stream = (yield of coarse stream from air classification × starch content of coarse stream, dry basis)/(starch content of pin‐milled flour, dry basis).

The protein content of the faba bean coarse stream decreased with soaking and germination from 193 to 151 g kg^−1^; however, germination duration did not affect the protein content. This trend further indicates that seed germination improved the separation of protein and starch during air classification, resulting in higher stream purity. In contrast, the protein content of the pea coarse stream was similar across all treatments (123–132 g kg^−1^). Residual protein in air‐classified coarse streams has been attributed to protein bodies adhering to starch granules, as well as agglomerates of starch and other cellular components.^32^ Skylas *et al*.^34^ also found that the coarse fraction of air classified pea had a lower protein content than that of faba bean (174 vs 235 g kg^−1^). The corresponding starch content reported in that study was 663 g kg^−1^ for pea and 617 g kg^−1^ for faba bean, which are slightly lower than the values reported in the current study.

The ash content of the coarse stream of pea remained stable (~14 g kg^−1^) until 96 h germination where it increased slightly to 18 g kg^−1^. For faba bean, the ash content of the coarse stream decreased after soaking and 24–48 h germination but then increased to similar levels as the control after 96 h of germination (Table 5), which closely mimics the ash content of pin‐milled faba bean flour. The observed variations in ash content could be attributed to changes in biochemical composition during germination.

## CONCLUSIONS

This study investigated the potential of germination as a pretreatment method to improve the efficiency and purity of air classification streams in pulse processing, specifically focusing on pea (CDC ‘Meadow’) and faba bean (DL ‘Rico’) seeds. The aim of the study was to generate scientific insights for optimizing pulse fractionation methodologies by investigating the structural modifications induced by germination and their subsequent impact on the air‐classification process. The results demonstrated that germination improved the air‐classification efficiency and the purity of the resulting streams significantly. Structural changes during germination facilitated the release of starch granules from the protein–fiber matrix, enhancing the separation efficiency of protein and starch. Notably, a gradual increase in separation efficiency was observed with prolonged germination, reaching optimal levels after 48 h of germination.

This emphasizes the importance of tailoring germination durations to maximize fractionation efficiency for different pulse types. An important outcome was that protein retention in the fine stream increased by 10.6% for pea and 25.6% for faba bean after 48 h of germination. As this study focused on the effects of seed germination on the processability of pea and faba bean flours by air classification, future work should evaluate the impact of germination pretreatment on nutritional attributes (e.g. antinutritional compounds and micronutrients), flavor, and functional properties of both coarse and fine fractions. Such insights can support the development of sustainable, high‐quality processing methods, addressing the growing demand for high quality pulse‐based ingredients in the food and feed industry.

## CONFLICT OF INTEREST

The authors declare no conflict of interest.

## AUTHOR CONTRIBUTIONS

Areha Abid: data curation, formal analysis, investigation, methodology, software, validation, visualization, writing – original draft. Kashika Sethi: data curation, formal analysis, investigation, methodology, software, validation, visualization. Andrea K. Stone: writing – original draft, writing – review and editing. Xialu Chen: data curation. Ke Ding: data curation. Thomas D. Warkentin: funding acquisition, resources, writing – review and editing. Bunyamin Tar'an: funding acquisition, resources, writing – review and editing. Yongfeng Ai: conceptualization, funding acquisition, methodology, project administration, resources, supervision, writing – review and editing. Michael T. Nickerson: conceptualization, funding acquisition, methodology, project administration, resources, supervision, writing – review and editing.

## Data Availability

The data that support the findings of this study are available from the corresponding author upon reasonable request.
